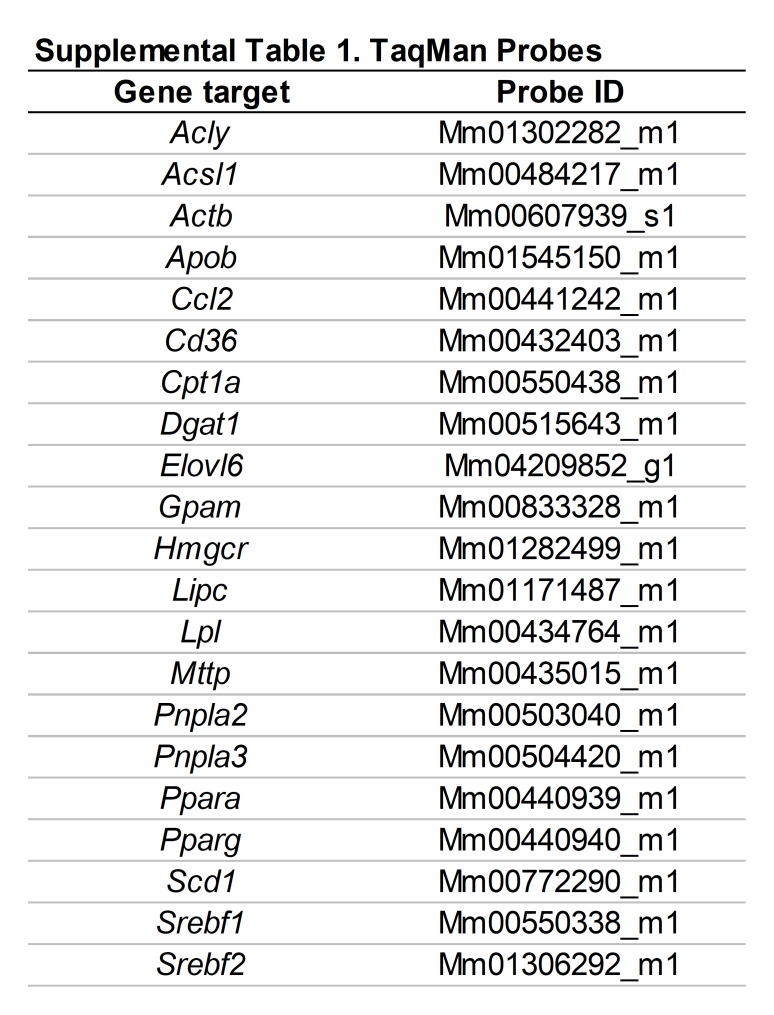# Dennd5b-Deficient Mice are Resistant to PCSK9-Induced Hypercholesterolemia and Diet-Induced Hepatic Steatosis

**DOI:** 10.1016/j.jlr.2022.100296

**Published:** 2022-10-13

**Authors:** Maura Mobilia, Callie Whitus, Alexander Karakashian, Hong S. Lu, Alan Daugherty, Scott M. Gordon

**Affiliations:** 1 Saha Cardiovascular Research Center, University of Kentucky, Lexington, KY, USA; 2 Department of Physiology, University of Kentucky, Lexington, KY, USA

**Keywords:** atherosclerosis, lipoproteins, cholesterol, dietary fat, lipids, liver, proprotein convertase subtilisin/kexin type 9 serine protease, triglyceride, hepatic steatosis, AAV, adeno-associated virus, FPLC, fast protein liquid chromatography, LDLR, low-density lipoprotein receptor, PCSK9, proprotein convertase subtilisin/kexin type 9

## Abstract

*Dennd5b* plays a pivotal role in intestinal absorption of dietary lipids in mice and is associated with body mass index in humans. This study examined the impact of whole-body *Dennd5b* deletion on plasma lipid concentrations, atherosclerosis, and hepatic lipid metabolism in mice. Hypercholesterolemia was induced in *Dennd5b*^*−/−*^ mice by infection with an adeno-associated virus expressing the proprotein convertase subtilisin/kexin type 9 serine protease (PCSK9) gain-of-function mutation (PCSK9D377Y) and feeding a Western diet for 12 weeks. Body weight and plasma lipid concentrations were monitored over 12 weeks, and then aortic atherosclerosis and hepatic lipid content were quantified. Compared to *Dennd5b*^*+/+*^ mice, *Dennd5b*^*−/−*^ mice were resistant to diet-induced weight gain and PCSK9-induced hypercholesterolemia. Atherosclerosis quantified by *en face* analysis and in aortic root sections, revealed significantly smaller lesions in *Dennd5b*^*−/−*^ compared to *Dennd5b*^*+/+*^ mice. Additionally, *Dennd5b*^*−/−*^ mice had significantly less hepatic lipid content (triglyceride and cholesterol) compared to *Dennd5b*^*+/+*^ mice. To gain insight into the basis for reduced hepatic lipids, quantitative PCR was used to measure mRNA abundance of genes involved in hepatic lipid metabolism. Key genes involved in hepatic lipid metabolism and lipid storage were differentially expressed in *Dennd5b*^*−/−*^ liver including *Pparg*, *Cd36*, and *Pnpla3*. These findings demonstrate a significant impact of *Dennd5b* on plasma and hepatic lipid concentrations and resistance to PCSK9-induced hypercholesterolemia in the absence of *Dennd5b*.

The intestine and liver play central roles in the maintenance of systemic lipid homeostasis. Dietary lipids are absorbed by the small intestine and packaged into chylomicrons for distribution to peripheral tissues via the circulation (1). The liver acts as a hub for both de novo synthesis and storage of lipid. During fasting conditions, the liver maintains systemic lipid supply by packaging and secretion of very low-density lipoproteins (VLDL) into plasma (2). Altered metabolic regulation or disruption of lipid homeostasis in either of these organs can influence plasma lipid concentrations and risk of atherosclerotic vascular disease or metabolic disease (e.g., obesity and hepatic steatosis) (3, 4).

Dietary lipid content can have a significant impact on hepatic lipid metabolism and plasma lipoprotein concentrations (5, 6, 7). We demonstrated recently a role for the gene *Dennd5b* in intestinal absorption of dietary lipid (8). *Dennd5b*^*−/−*^ mice have impaired absorption of ingested triglyceride due to impaired intestinal chylomicron secretion by enterocytes. As a consequence, *Dennd5b*^*−/−*^ mice are resistant to diet-induced obesity, hypercholesterolemia, and atherosclerosis (8). From these studies, it is unclear if the protective effect of *Dennd5b* deficiency on atherosclerosis is a direct consequence of altered plasma cholesterol concentrations or other indirect effects of *Dennd5b*. The goal of the current study was to examine the effects of *Dennd5b* on plasma lipoprotein concentrations, hepatic lipid metabolism, and atherosclerosis in a model of hypercholesterolemia that is not strictly diet-induced.

Proprotein convertase subtilisin/kexin type 9 (PCSK9) is an endogenous regulator of plasma low density lipoprotein associated cholesterol (LDL-C) by facilitating degradation of hepatic LDL receptor (LDLR) (9). Higher plasma PCSK9 concentrations result in reduced hepatic LDLR and increased plasma LDL-C concentrations. In humans, a gain-of-function *PCSK9* variant (D374Y) confers increased LDL-C and increased risk of atherosclerotic vascular disease, while loss-of-function mutations have the opposite effect (10, 11, 12). Adeno-associated virus (AAV)-mediated overexpression of the mouse PCSK9 gain-of-function analog (D377Y) has been used to induce hypercholesterolemia in mice (13, 14, 15, 16). We hypothesized that reduced absorption of dietary lipids in *Dennd5b*^*−/−*^ mice would result in peripheral effects on lipid metabolism and atherosclerotic vascular disease but that PCSK9-induced hypercholesterolemia would overcome the protective effect of *Dennd5b* deficiency. To test this hypothesis, *Dennd5b*^*−/−*^ mice were fed Western diet and administered AAV to express the mouse PCSK9 gain-of-function variant D377Y to facilitate induction of hypercholesterolemia. Our findings reveal that *Dennd5b*^*−/−*^ mice are resistant to PCSK9-induced hypercholesterolemia and atherosclerosis. Furthermore, we observed a significant impact of *Dennd5b* on diet-induced hepatic steatosis which may be mediated by effects of *Dennd5b* on expression of genes regulating hepatic lipid metabolism.

## Materials and Methods

### Mouse housing and diets

*Dennd5b*^*+/+*^*and*^*−/−*^ mice were generated as described previously (8) and maintained at the University of Kentucky Division of Laboratory Animals Resources in individually vented cages (max. 5 mice per cage) on a 14:10 h (light:dark) cycle and maintained at 22°C (72°F). Teklad sani-Chip (#7090A, Harlan Teklad) bedding is used in cages and colonies are maintained on a standard rodent diet (#2918 Envigo) with *ad libitum* access to food and water. All studies were performed in male mice. Beginning the day of AAV administration, mice were switched to a Western diet (21% fat + 0.2% cholesterol; TD.88137, Envigo) and maintained on this diet for 12 weeks. All animal studies were approved by the University of Kentucky Institutional Animal Care and Use Committee.

### PCSK9 gain-of-function AAV administration

Mouse PCSK9 (D377Y) gain-of-function AAV vector (serotype 8) was used to induce hypercholesterolemia by hepatic overexpression of PCSK9, as reported previously (13). The AAV vectors express mouse PCSK9D377Y (analogous to the human PCSK9D374Y gain-of-function mutation) or an empty AAV vector was used as a control. AAV (2 × 10^11^ GC/mouse) was diluted in sterile PBS at 200 μl and administered by intraperitoneal injection on study day one.

### Blood collection, plasma lipid analysis, and PCSK9 measurement

Blood was collected from nonfasting mice by retro-orbital bleeding using 250 μl heparinized glass capillary tubes. Collections were performed in the early morning, at the beginning of the light phase. Plasma was obtained by centrifugation at 1,250 *g* for 10 min at 4°C. Colorimetric enzymatic assays were used to measure plasma lipid concentrations: Cholesterol-E (# 999-02601, FujiFilm) and L-Type Triglyceride M (994-02891 and 990-02991, FujiFilm). HDL-C was measured in plasma after polyethylene glycol precipitation of apoB-containing lipoproteins (17). Non-HDL cholesterol (Non-HDL-C) was calculated by subtraction of HDL-C from total cholesterol. As a secondary approach to examine lipid distribution among lipoprotein classes, Fast Protein Liquid Chromatography (FPLC) separation of plasma lipoproteins was performed on Atka Pure instrument with one Superose 6 Increase column (Cytiva). Plasma (100 μl, pooled with equal volumes contributed from all animals within each group) was injected onto the column and eluted with PBS at a flow rate of 0.5 ml/min. Fractions (0.5 ml/fraction) were collected in deep-well 96 well plates, and total cholesterol and triglyceride were measured across fractions with enzymatic assays. Concentrations of plasma PCSK9 protein were measured on plasma collected at the end of the study (week 12) using a Quantikine ELISA kit (R&D Biosystems; #MPC900) according to manufacturer’s instructions.

### Quantification of atherosclerosis

After 12 weeks feeding on Western diet, mice were sacrificed by injectable anesthesia overdose (ketamine 210 mg/kg and xylazine 30 mg/kg) and were perfused with PBS via the left ventricle after severing the right renal artery. Mouse aortas were harvested and fixed in 10% neutral buffered formalin for 24 h at room temperature, then stored in PBS at 4°C. Aortas were cleaned thoroughly by removal of periaortic adventitia, stained with Oil Red O, and cut open and pinned flat for *en face* analysis. Images were taken on Nikon Imaging Software and ImageJ was used to quantify stained plaque areas. Lesion area was calculated by (lesion area/total aortic surface area) ∗100. Aortic roots were embedded in optimal cutting temperature compound immediately after harvest and kept at −80°C for frozen sectioning. Serial sections (10 um) were cut and placed on slides (Thermo Fisher Scientific). For quantification of area of neutral lipids, sections were fixed in fresh 4% paraformaldehyde for 10 min. Root sections were then stained with Oil Red O and imaged on a Zeiss AxioScan slide scanner. Lesion quantification was performed using Zen software (Zeiss) and is presented as lesion area in mm^2^.

### Hepatic histology and lipid analysis

Liver tissue was harvested and fixed in 10% formalin overnight at room temperature and then placed in 30% sucrose for 24 h at 4°C before embedding in optimal cutting temperature compound and stored at −20°C until sectioning on a cryostat. Sections (10 μm) were cut and placed on slides. Liver sections were then stained with Oil Red O, mounted in gelatin, and imaged immediately using Nikon Elements imaging software. Lipids were extracted from snap-frozen liver tissue by chloroform methanol extraction. After drying under nitrogen, the mass of extracted lipids from the chloroform phase was measured and used to calculate total liver lipid as a percent of original tissue mass. Extracted lipids were dissolved in 1 ml of chloroform + 1% Triton, dried down again, resuspended in 500 μl of nuclease free water, and vortexed vigorously before lipids were quantified by enzymatic assays.

### Hepatic protein analysis

At harvest, perfused livers were snap frozen in liquid nitrogen and stored at −80°C until protein extraction. Liver tissue (∼30 mg) was placed in RIPA lysis buffer (Thermo, #89901) containing 1x HALT (Thermo, #78442) and homogenized in red Rino tubes (Next Advance) using a Bullet Blender (Next Advance, model #BB24-AU) at full speed for 5 min. Lysate was centrifuged at 10,000 *g* for 10 min at 4°C and supernatant was collected. Bicinchoninic acid protein assay was performed for quantification of total protein. Lysates were used for Western blotting to evaluate PCSK9-induced reduction of hepatic LDLR protein (R&D Biosystems, #AF2255) using *Actb* (Sigma #A5441) for normalization.

### Hepatic gene expression

At harvest, mouse liver tissue was placed in 1.5 ml tubes containing RNAlater (Invitrogen) and stored at 4°C. Total RNA was isolated using RNAqueous-4PCR kit (Invitrogen) and stored at −80°C. Complementary DNA was produced using High Capacity Complementary DNA Reverse Transcription Kit (Applied Biosystems) in a 20 μl reaction. For gene expression analysis, TaqMan gene expression assays (Applied Biosystems) were used (assay probe catalog numbers provided in supplemental Table S1). All targets were normalized to beta-actin expression.

### VLDL secretion assay

Mice were fasted for 4 h, then injected with Triton WR-1339 (Tyloxapol) in saline (15% wt:vol). Blood was collected at baseline, then 1, 2, and 4 h after injection. Blood was centrifuged immediately after each collection at 1,250 *g* for 10 min at 4°C. Plasma was collected and assayed for triglyceride concentrations using colorimetric enzymatic assays (Wako Diagnostics). Western blotting for apoB was performed using anti-apoB antibody (Abcam #20737).

### Statistical analysis

Statistical comparisons between two groups were performed by unpaired Student’s *t*-test. When comparisons involved more than two groups, analyses were performed by ANOVA with posthoc adjustment using methods indicated for each experiment in figure legends. Statistical comparisons were performed using GraphPad Prism software. For all experiments, *P* values < 0.05 were considered statistically significant. Principal components and multivariate regression analyses of gene expression data were performed using JMP Genomics version 10.2.

## Results

### PCSK9 expression delayed Western diet-induced weight gain in wild type mice

**Fig. 1:**
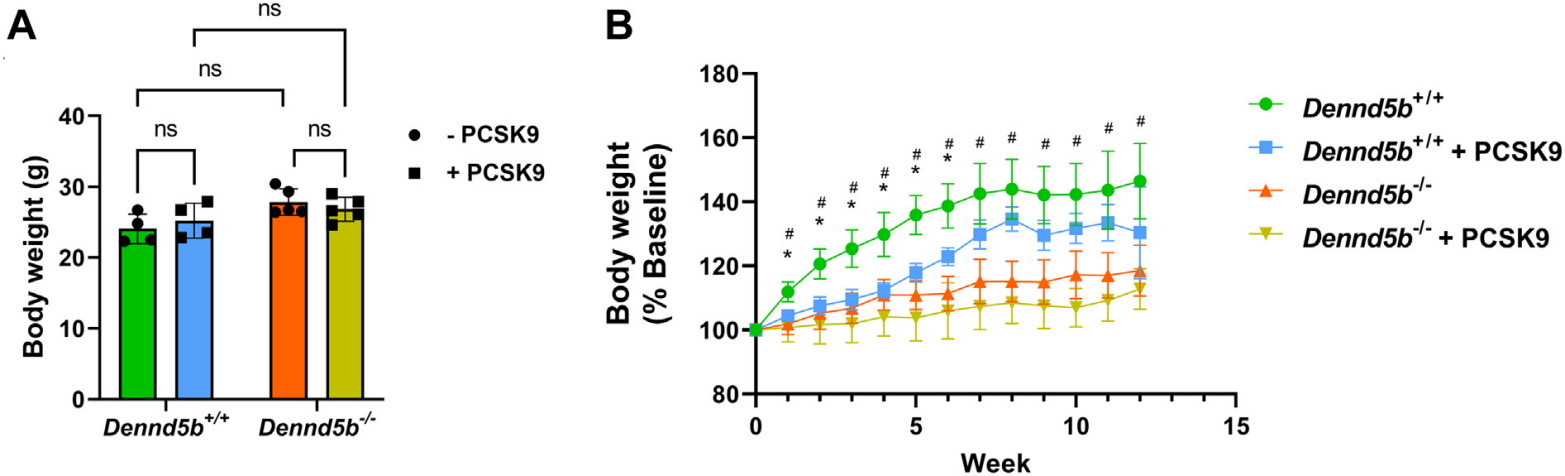
*Dennd5b*^*−/−*^ were resistant to diet-induced weight gain regardless of PCSK9 gain-of-function expression. A: Baseline body weights. B: Changes in body weights of wild type and *Dennd5b*^*−/−*^ mice during 3-months feeding a Western diet. Body weights were measured weekly throughout the study. *Dennd5b*^*+/+*^ n = 4/group and *Dennd5b*^*−/−*^ n = 5/group. Statistical comparisons by two-way ANOVA with Tukey correction for multiple comparisons. ∗*P* < 0.05 for *Dennd5b*^+/+^ versus *Dennd5b*^+/+^ + PCSK9. # *P* < 0.05 for *Dennd5b*^+/+^ versus *Dennd5b*^*−/−*^. PCSK9, proprotein convertase subtilisin/kexin type 9 serine protease; PCSK9, proprotein convertase subtilisin/kexin type 9.

To induce hypercholesterolemia, wild type and *Dennd5b*^*-/-*^ mice received intraperitoneal injection of AAV to drive hepatic expression of the mouse PCSK9 gain-of-function variant. Mice were fed Western diet beginning immediately after receiving AAV injection. Mice remained on Western diet for 12 weeks. To evaluate weight gain between groups, mice were weighed weekly. All groups had similar baseline body weights (Fig. 1A). *Dennd5b*^*−/−*^ were resistant to Western diet-induced weight gain observable from week 1 on diet (Fig. 1B) as reported previously (8). PCSK9 overexpression did not impact body weight in *Dennd5b*^*−/−*^ mice; however, wild type mice receiving PCSK9 demonstrated a delayed gain of body weight compared to mice not infected with PCSK9 AAV (Fig. 1B). Following the delay in weight gain during the initial seven weeks, weight gain became equivalent in wild type mice not infected with PCSK9 AAV.

### Plasma PCSK9 concentrations and hepatic LDLR protein abundance

**Fig. 2:**
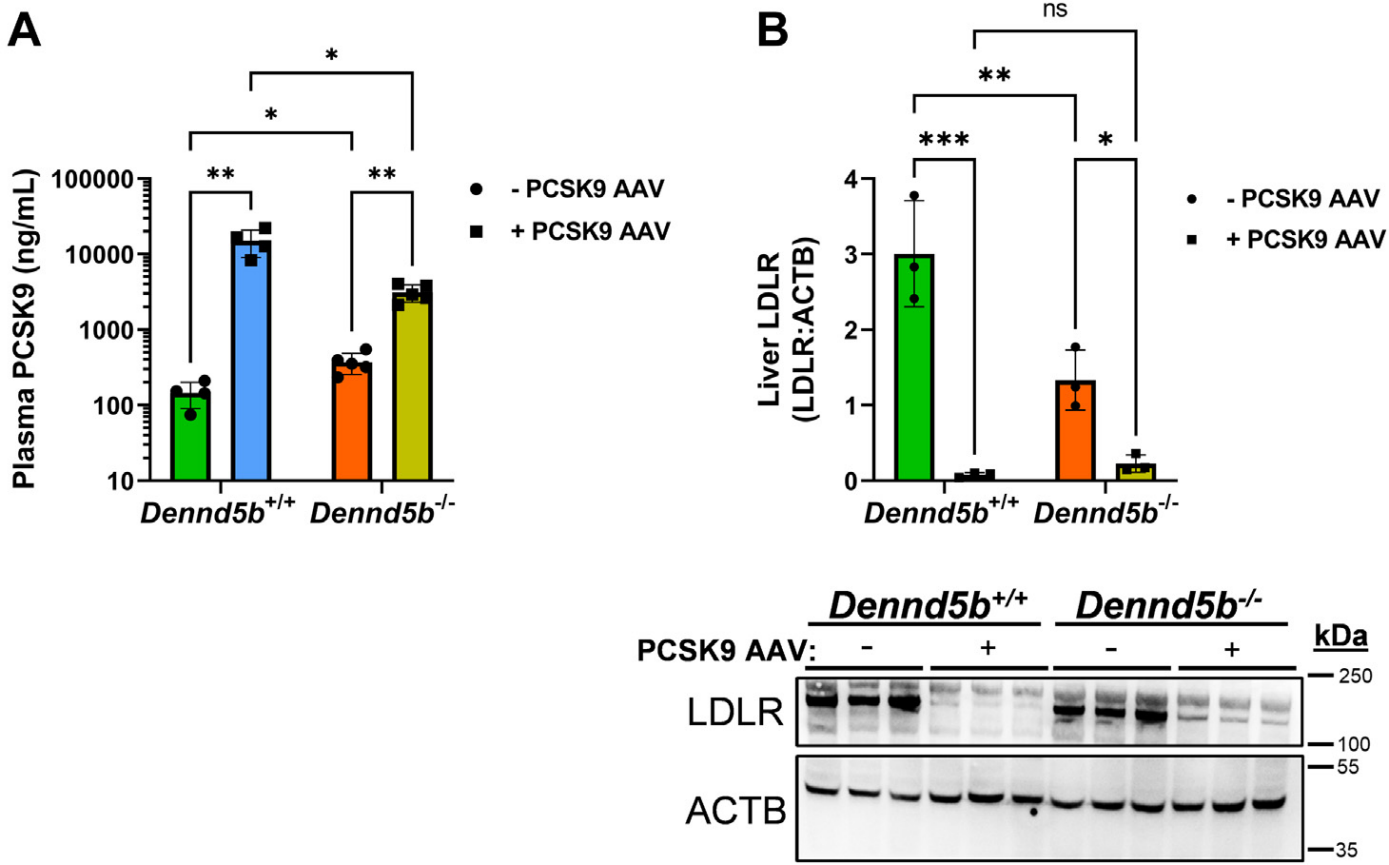
*Dennd5b*^*−/−*^ affects plasma PCSK9 concentrations and hepatic LDLR abundance when fed a Western diet. A: PCSK9 was measured by ELISA in wild type and *Dennd5b*^*−/−*^ mouse plasma collected on week 12. B: Hepatic LDLR protein was determined by Western blot on lysates from snap frozen tissue harvested on week 12. Statistical comparisons were performed by two-way ANOVA with Tukey correction for multiple comparisons. ∗*P* < 0.05, ∗∗*P* < 0.01, ∗∗∗*P* < 0.001. LDLR, low density lipoprotein receptor; PCSK9, proprotein convertase subtilisin/kexin type 9.

Plasma concentrations of PCSK9 protein were measured on week 12 in wild type and *Dennd5b*^*-/-*^ mice that were or were not infected with PCSK9 AAV. In the absence of PCSK9 AAV, *Dennd5b*^*-/-*^ mice had nearly three times higher plasma concentrations of PCSK9 than wild type (369.3 vs. 145.2 ng/ml; *P* < 0.05; Fig. 2A). Consistent with this finding, *Dennd5b*^*−/−*^ mice have lower hepatic LDLR protein (3.0 vs. 1.3 LDLR:ACTB band ratio, *P* < 0.01) detected by Western blotting (Fig. 2B). AAV infection significantly increased plasma PCSK9 concentrations in wild type and *Dennd5b*^*−/−*^ mice, although to a lesser extent in *Dennd5b*^*−/−*^ mice (14,931 vs. 3,103 ng/ml, *P* < 0.01; Fig. 2A). Despite this difference in plasma PCSK9 response to AAV-mediated induction, both genotypes experienced nearly complete reduction of LDLR protein (0.08 vs. 0.23 LDLR:ACTB band ratio, *P* = 0.97; Fig. 2B).

### *Dennd5b*^*−/−*^ mice were resistant to PCSK9-induced hypercholesterolemia

**Fig. 3:**
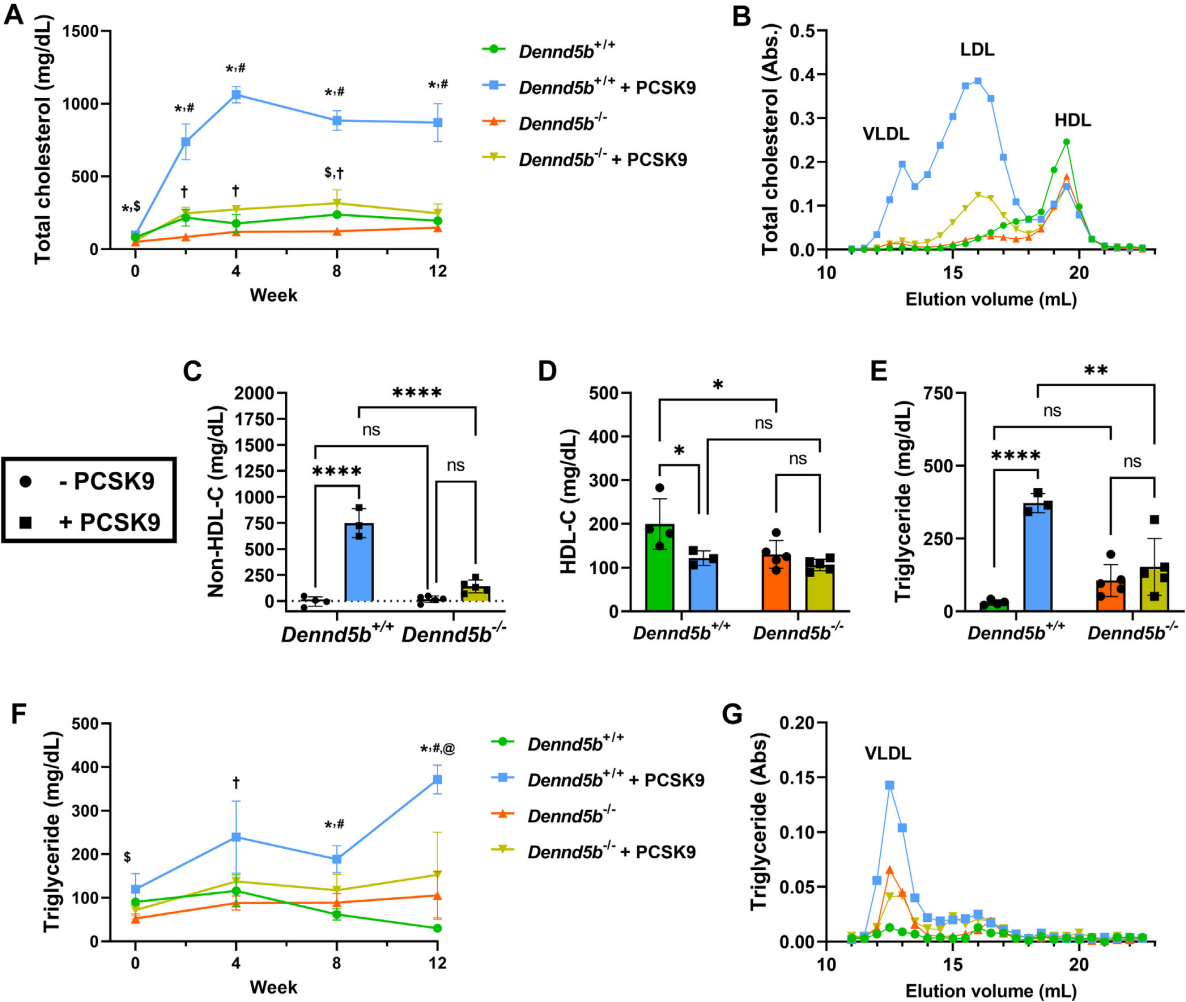
*Dennd5b*^*−/−*^ mice were resistant to PCSK9D377Y-induced hyperlipidemia. A: Total plasma cholesterol concentration measurements during the study. B: Fast-Protein Liquid Chromatography (FPLC) lipoprotein panel on cholesterol from week 12 plasma. C: Plasma non-HDL cholesterol was calculated from total cholesterol–HDL cholesterol. D: HDL cholesterol was measured in plasma after depletion of apoB-containing lipoproteins by polyethylene glycol precipitation. E: Plasma triglyceride concentrations on week 12. F: Plasma triglyceride measurements during the study. G: FPLC triglyceride profiles on plasma from week 12. Statistical comparisons for (A, F) were performed by mixed effects analysis with the Geisser-Greenhouse correction. Symbols indicate *P* < 0.05 for: ^$^*Dennd5b*^+/+^ versus *Dennd5b*^*−/−*^; ^#^*Dennd5b*^*+/+*^ versus *Dennd5b*^*+/+*^ + PCSK9; ∗ *Dennd5b*^*+/+*^ + PCSK9 versus *Dennd5b*^*−/−*^ + PCSK9; ^†^*Dennd5b*^*−/−*^ versus *Dennd5b*^*−/−*^ + PCSK9. Statistical comparisons for C, D, E were performed by two-way ANOVA with Tukey correction for multiple comparisons. ∗*P* < 0.05, ∗∗*P* < 0.01, ∗∗∗∗*P* < 0.0001. FPLC, fast protein liquid chromatography; PCSK9, proprotein convertase subtilisin/kexin type 9.

To evaluate plasma lipid concentrations, plasma samples were collected at baseline (week 0), 2, 4, 8, and 12 weeks postinfection. Total cholesterol and triglyceride concentrations were measured at each interval by lipid assay, and lipoprotein profiles were resolved by FPLC on plasma collected at the end of the study. PCSK9 overexpression in wild type mice significantly increased plasma total cholesterol concentrations that plateaued at week 4 (+500% compared to wild type controls) and sustained through week 12 (Fig. 3A). PCSK9 had a more modest impact on total plasma cholesterol concentrations in *Dennd5b*^*−/−*^ mice (+128% compared to *Dennd5b*^*−/−*^ controls on week 4). To examine lipoprotein profiles, FPLC was performed to revolve the major lipoprotein classes. The PCSK9-induced cholesterol increase was predominantly due to increased LDL in wild type and *Dennd5b*^*−/−*^ mice (Fig. 3B). Wild type mice also had a larger VLDL peak that was not present in *Dennd5b*^*−/−*^ plasma. Consistent with the FPLC profile, plasma non-HDL-C was significantly increased by PCSK9 overexpression in wild type (*P* < 0.0001) and more modest elevation was observed in *Dennd5b*^*−/−*^ (*P* < 0.07) mice (Fig. 3C). As reported previously, *Dennd5b*^*−/−*^ mice had lower HDL-C concentrations than wild type (Fig. 3D) (8). PCSK9 overexpression reduced HDL-C in wild type mice but did not affect HDL-C in *Dennd5b*^*−/−*^.

At baseline, *Dennd5b*^*−/−*^ mice had lower plasma triglyceride concentrations (90.4 vs. 52.5 mg/dl, *P* < 0.01). In wild type mice, PCSK9 induced an increase in plasma triglyceride concentrations that was most prominent on week 12 (Fig. 3E, F). Plasma triglyceride was not affected by PCSK9 in *Dennd5b*^*−/−*^ mice. Effects on plasma triglyceride were predominantly associated with the VLDL peak of the FPLC profile (Fig. 3G).

### *Dennd5b*^*-/-*^ mice were resistant to PCSK9-induced atherosclerosis

**Fig. 4:**
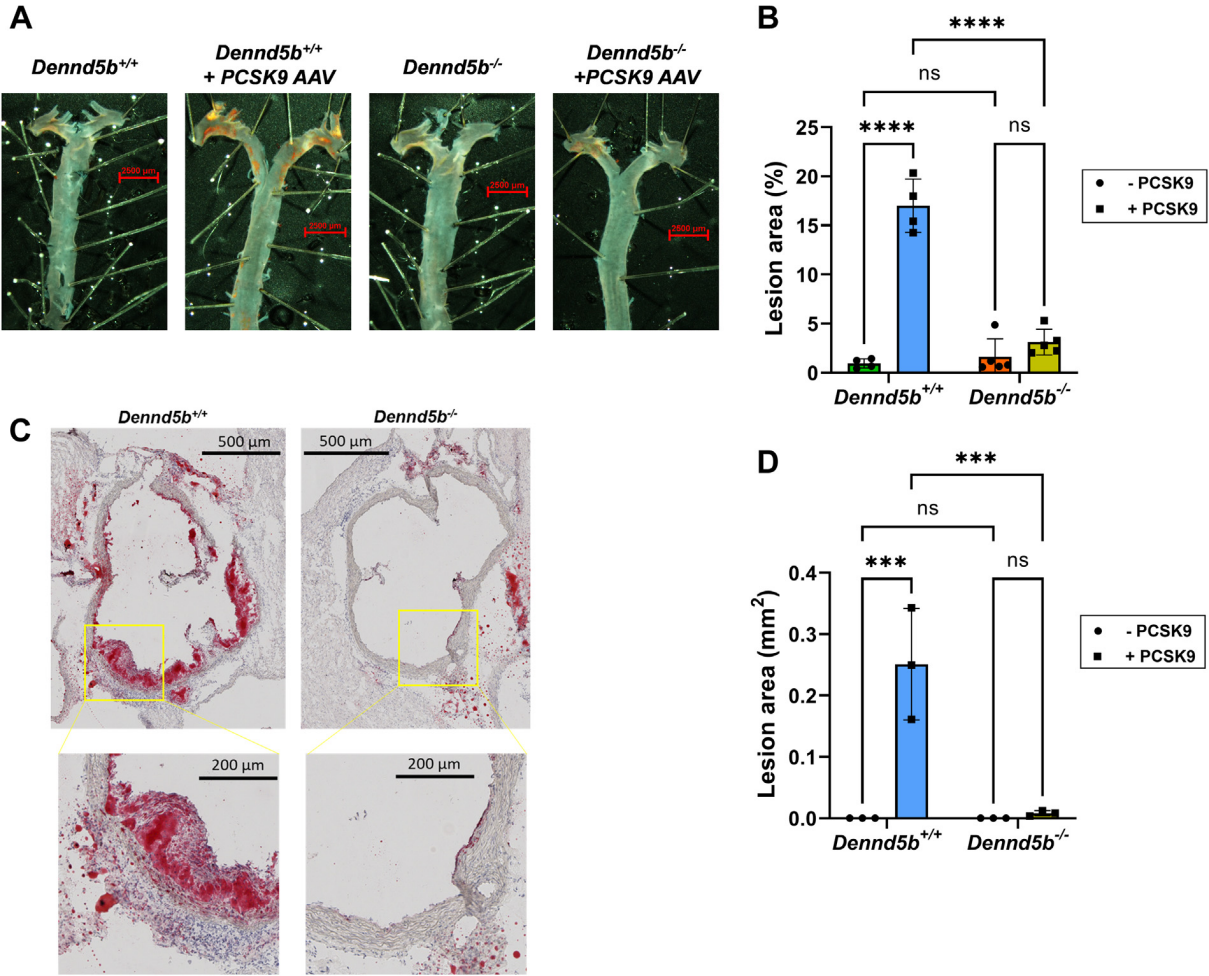
*Dennd5b*^*−/−*^ mice were resistant to PCSK9-induced atherosclerosis. A: Whole aortas stained with Oil Red O for *en face* analysis of plaque area. B: Quantification of plaque lesion area as a percentage of the entire aorta. C: Aortic root serial sections cut at 10 μm and Oil Red O stained. Only sections from PCSK9-induced Dennd5b+/+ and −/− mice are displayed. D: Quantification of plaque lesion area of aortic root in mm^2^. Statistical comparisons were performed by two-way ANOVA with Tukey correction for multiple comparisons. ∗∗∗*P* < 0.001, ∗∗∗∗*P* < 0.0001. PCSK9, proprotein convertase subtilisin/kexin type 9.

**Fig. 5:**
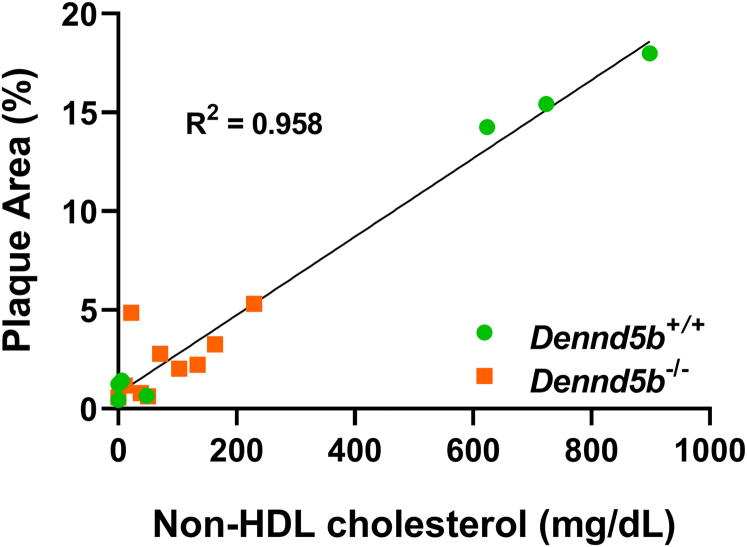
Correlation between non-HDL-C concentrations and lesion area. Linear regression analysis of plasma non-HDL-C concentrations on week 12 and lesion area from *en face* analysis of aortas from wild type and *Dennd5b*^*−/−*^ mice. Data include mice with and without PCSK9 overexpression. PCSK9, proprotein convertase subtilisin/kexin type 9.

After 12 weeks of feeding a Western diet, aortas were harvested and stained with Oil Red O for quantification of atherosclerotic plaque. *En face* analysis revealed an increase in lesion area in wild type mice infected with PCSK9 AAV compared to wild type controls fed a Western diet (17% vs. 0.9% lesion area, *P* < 0.0001) (Fig. 4A, B). However, *Dennd5b*^*−/−*^ + PCSK9 had significantly smaller lesions size than *Dennd5b*^*+/+*^ + PCSK9 (3.1% vs. 17%, *P* < 0.0001). Analysis of lesion area in aortic roots from the same mice revealed a similar pattern of lower lesion area in *Dennd5b*^*−/−*^ + PCSK9 (Fig. 4C, D). These results demonstrate that *Dennd5b*^*-/-*^ mice were resistant to PCSK9-induced atherosclerosis in vivo*.* This is consistent with the lower non-HDL-C concentrations in *Dennd5b*^*−/−*^ + PCSK9 mice. Analysis of the relationship between non-HDL-C concentrations and *en face* lesion area demonstrated that *Dennd5b*^*−/−*^ mice did not deviate from the expected linear relationship between these measures (Fig. 5). This would suggest that the protective effect of *Dennd5b*^*−/−*^ on atherosclerosis is likely simply due to lower plasma non-HDL-C concentrations.

### *Dennd5b*^*−/−*^ mice were resistant to diet-induced hepatic steatosis

**Fig. 6:**
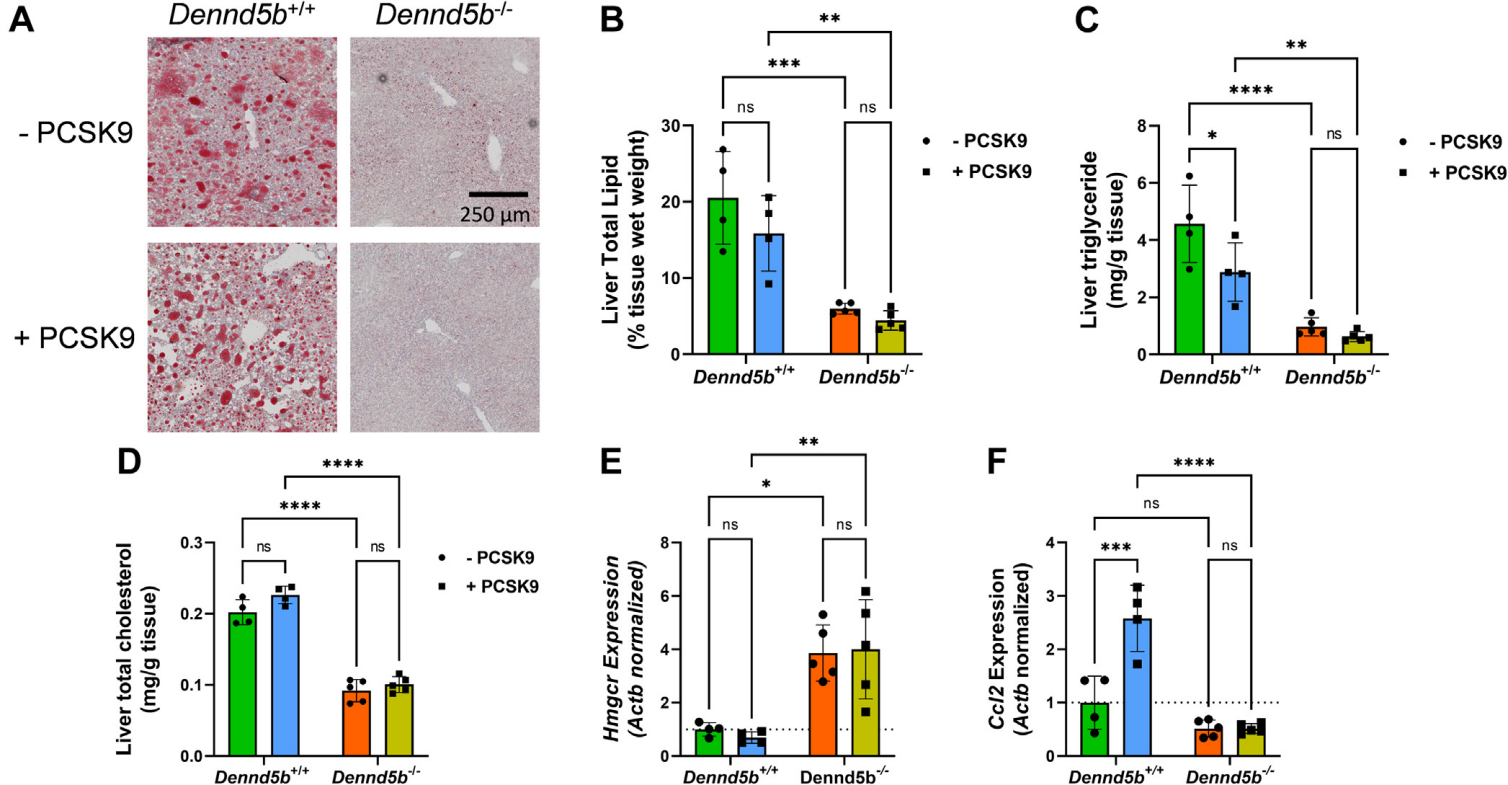
*Dennd5b*^*−/−*^ mice were resistant to diet-induced hepatic steatosis. A: Images of liver (10 μm) sections Oil Red O stained at 20× magnification. B–D: Lipids were extracted from liver after 12 weeks of feeding a Western diet and total triglyceride and cholesterol concentrations were measured by enzymatic assay. Quantitative polymerase chain reaction quantification of *Hmgcr* (E) and *Ccl2* (F) mRNA abundance in liver normalized to beta-actin. Statistical comparisons were performed by two-way ANOVA with Tukey correction for multiple comparisons. ∗*P* < 0.05, ∗∗*P* < 0.01, ∗∗∗*P* < 0.001, ∗∗∗∗*P* < 0.0001.

Upon gross anatomical examination, it was clear that wild type mice had developed hepatic steatosis (visually observed as a pale liver). However, livers from *Dennd5b*^*−/−*^ mice appeared considerably less pale and maintained a dark reddish-brown color. To examine hepatic lipid accumulation, liver sections were prepared and stained with Oil Red O. Considerably, greater lipid accumulation was observed in wild type livers than *Dennd5b*^*−/−*^ (Fig. 6A). Total hepatic lipid content was lower in *Dennd5b*^*−/−*^ livers independent of PCSK9 overexpression (Fig. 6B). Lower triglyceride and cholesterol contributed to the lower lipid content in *Dennd5b*^*−/−*^ livers (Fig. 6C, D). PCSK9 overexpression reduced hepatic triglyceride content in wild type mice; this was not observed in *Dennd5b*^*−/−*^ mice. PCSK9 overexpression had little impact on hepatic cholesterol in wild type or *Dennd5b*^*−/−*^ mice. Although hepatic cholesterol content was lower in *Dennd5b*^*−/−*^ mice, we observed a four-fold higher abundance of *Hmgcr mRNA*, the rate limiting enzyme in cholesterol synthesis, in these mice (Fig. 6E). In addition to reduced lipid content, *Dennd5b*^*−/−*^ mice exhibited lower abundance of *Ccl2* mRNA than wild type mice, suggestive of lower levels of hepatic inflammation (Fig. 6F).

### *Dennd5b*^*-/-*^ affected abundance of genes involved in hepatic lipid metabolism

**Fig. 7:**
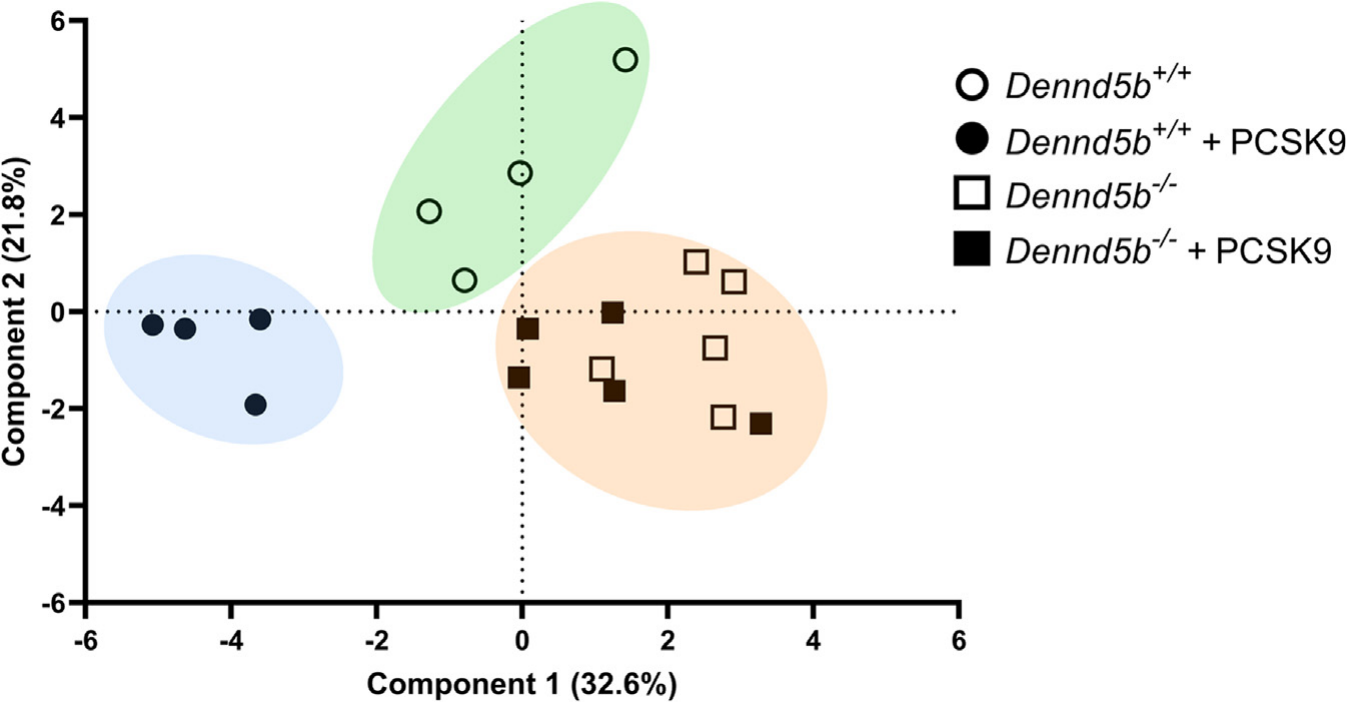
Distinct hepatic gene expression signature in *Dennd5b*^*−/−*^ mice. Principal components analysis was performed on RT-qPCR data from 20 genes with known roles in hepatic lipid metabolism from mouse liver tissue.

**Fig. 8:**
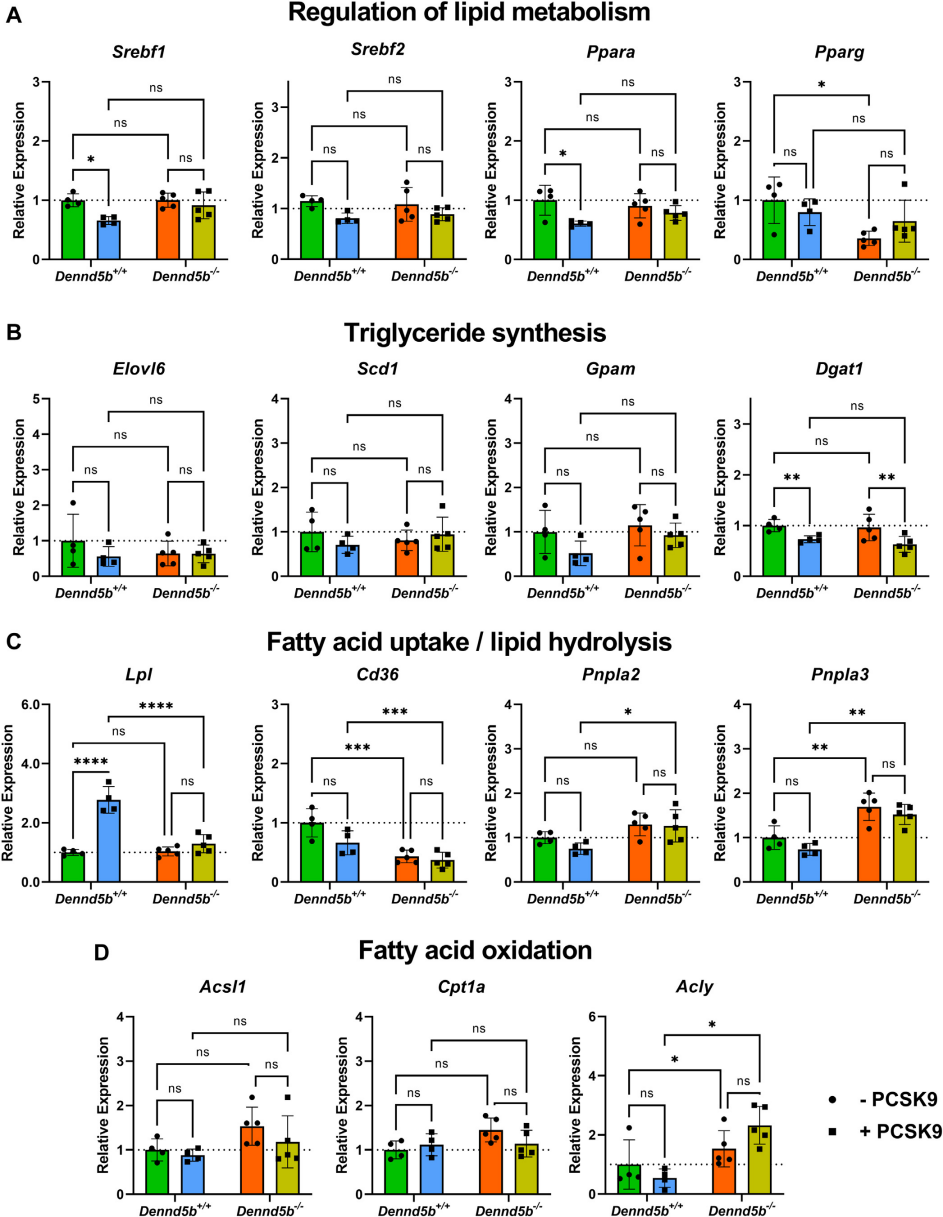
*Dennd5b*^*-/-*^ affected mRNA abundance of genes involved in hepatic lipid metabolism. A–D: Liver mRNA abundance was measured by qPCR normalized to beta-actin after 12 weeks on western diet in wild type and *Dennd5b*^*−/−*^ mice – PCSK9 AAV (circles) or + PCSK9 AAV (squares). Statistical comparisons were performed by two-way ANOVA with Tukey correction for multiple comparisons. ∗*P* < 0.05, ∗∗*P* < 0.01, ∗∗∗*P* < 0.001, ∗∗∗∗*P* < 0.0001. AAV, adeno-associated virus; PCSK9, proprotein convertase subtilisin/kexin type 9.

**Fig. 9:**
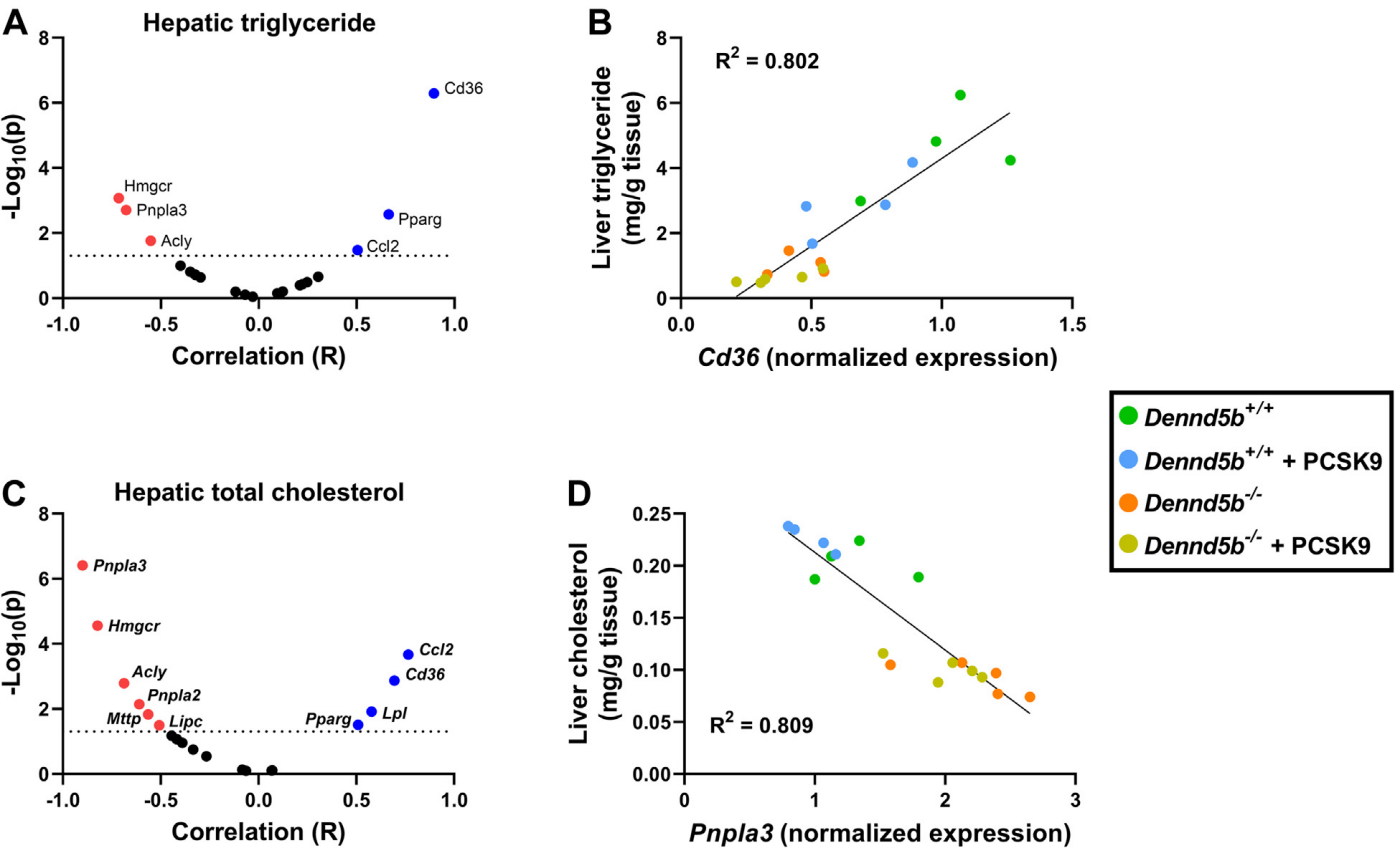
Correlation between hepatic lipids and gene expression. A: Multivariate regression analysis was performed to examine relationships between hepatic triglyceride content and expression of 20 genes involved in hepatic lipid metabolism. B: Scatterplot displaying the correlation for the gene most strongly correlated with hepatic triglyceride. C, D: A similar analysis was performed for hepatic total cholesterol.

To gain insight into the mechanistic basis for reduced hepatic lipid content in *Dennd5b*^*−/−*^ mice, mRNA abundance of 20 genes involved in several aspects of lipid metabolism were measured by quantitative PCR (supplemental Table S1). Principal components analysis of gene abundance data revealed three distinct clusters that segregated *Dennd5b*^*−/−*^ from wild type mice and further revealed expression profile differences induced by PCSK9 AAV in wild type but not in *Dennd5b*^*−/−*^ livers (Fig. 7). Measurement of several transcriptional regulators of lipid metabolism revealed significantly lower abundance of *Pparg* mRNA in *Dennd5b*^*−/−*^ mice (Fig. 8A), a gene known to regulate FA storage and glucose metabolism in several tissues. mRNA abundance for other lipid regulator genes (*Srebf1, Srebf2, Ppara*) were not affected by *Dennd5b* genotype. However, *Srebf1* and *Ppara* transcript abundances were reduced by PCSK9 AAV treatment in wild type but not in *Dennd5b*^*−/−*^ mice. Genes involved in triglyceride synthesis were also not affected by *Dennd5b* genotype (Fig. 8B), although PCSK9 AAV infection reduced Dgat1 mRNA abundance in both genotypes. On the other hand, abundance of *Cd36* mRNA, encoding a protein involved in cellular FA uptake, was significantly lower in *Dennd5b*^*−/−*^ livers, and *Pnpla3*, encoding a protein involved in lipid droplet hydrolysis, was significantly increased in *Dennd5b*^*−/−*^ livers (Fig. 8C). Interestingly, hepatic *Lpl* transcript levels were significantly higher in PCSK9 AAV infected wild type mice but not in *Dennd5b*^*-/-*^ mice. mRNA abundance of genes involved in FA metabolism, particularly *Acly*, also tended to be higher in the *Dennd5b*^*−/−*^ mice (Fig. 8D). To gain insight into which of these gene expression effects had the greatest impact on hepatic triglyceride content, multivariate regression analysis was performed (Fig. 9A). *Cd36* stood out as having the greatest impact, showing a strong positive linear relationship between mRNA abundance and hepatic triglyceride content (Fig. 9B). A similar analysis was performed for hepatic total cholesterol (Fig. 9C). Interestingly, *Pnpla3* was the strongest associated gene, displaying a negative association with hepatic cholesterol (Fig. 9D).

### Increased VLDL secretion in *Dennd5b*^*-/-*^ mice

**Fig. 10:**
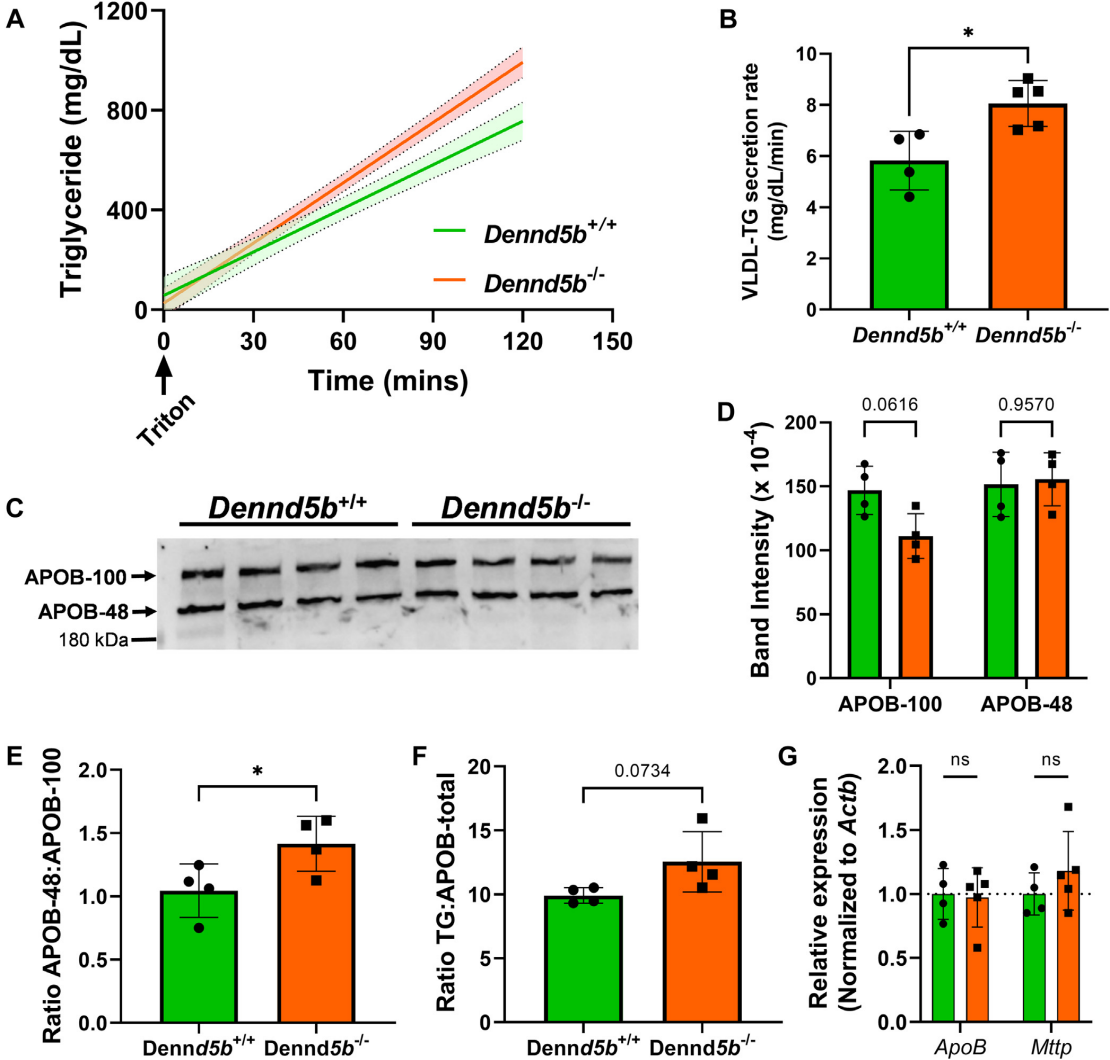
Hepatic VLDL secretion in *Dennd5b*^*−/−*^ mice. A, B: VLDL secretion rate was measured after injection of tyloxapol in fasting mice and measuring plasma triglyceride concentrations over time. C: APOB-100 and APOB-48 concentrations evaluated by Western blotting on plasma collected from VLDL secretion assay. D: Densitometry quantification of APOB-100 and APOB-48 band intensities. E: Ratio of APOB-100 to APOB-48 protein levels in wild type and *Dennd5b*^*−/−*^ mice. F: Ratio of total plasma triglyceride concentrations to total APOB protein. G: Relative mRNA abundance of *Apob* and *Mttp* measured by qPCR and normalized to beta-actin. Statistical comparisons by Student’s *t* test (B, E, F) or two-way ANOVA with Sidak correction (D, G). ∗*P* < 0.05.

To evaluate the effect of *Dennd5b* deficiency on hepatic VLDL production, we measured plasma triglyceride concentrations of fasting mice after inhibition of systemic lipase activity by intravenous injection of nonionic detergent (18). *Dennd5b*^*−/−*^ mice had a modest increase in the rate of triglyceride accumulation in plasma compared to wild type (Fig. 10A, B). Despite increase of plasma triglyceride concentrations, *Dennd5b*^*−/−*^ plasma had similar total APOB protein mass determined by Western blotting (Fig. 10C, D). Although changes in APOB isoform masses were not significant, the ratio of APOB-48:APOB-100 was significantly higher in *Dennd5b*^*−/−*^ plasma (Fig. 10E), suggesting altered *Apob* mRNA editing by *Apobec* or an effect on secretory processing of VLDL particles containing different isoforms. To further examine the quality of secreted VLDL, the ratio of triglyceride to total APOB was calculated, revealing a trend toward higher triglyceride content per VLDL particle in *Dennd5b*^*−/−*^ (Fig. 10F). These data suggest that increased triglyceride secretion in VLDL may contribute modestly to the reduced hepatic lipid content in *Dennd5b*^*−/−*^ mice*.* These findings do not appear to be mediated by changes in hepatic expression of genes related to VLDL assembly (Fig. 10G).

## Discussion

Intestinal absorption of dietary lipid and regulation of circulating lipoprotein concentrations by the liver are critical for the maintenance of systemic lipid homeostasis. We reported previously that *Dennd5b*^*−/−*^ mice exhibit reduced absorption of dietary triglyceride due to impaired chylomicron secretion by enterocytes. The current study aimed to examine the effects of *Dennd5b*^*−/−*^ on plasma lipoproteins, atherosclerosis, and hepatic lipid metabolism under conditions of induced hypercholesterolemia in mice fed a Western diet and infected with an AAV-inducing hepatic overexpression of the PCSK9 gain-of-function variant D377Y. Our findings reveal several new insights into the systemic effects of *Dennd5b* deficiency on lipid metabolism beyond our previous studies focused on the intestine.

Consistent with our previous report, *Dennd5b*-deficient mice were resistant to Western diet-induced weight gain. PCSK9 overexpression resulted in a delayed weight gain in wild type mice. To our knowledge, this effect of PCSK9-AAV on body weight in mice fed a Western diet has not been previously reported. This observation may be explained by studies in mice demonstrating that PCSK9 can limit adipogenesis by regulation of VLDLR protein abundance in adipose tissue (19). However, these findings may not be consistent in humans, as some small human studies have found that plasma PCSK9 concentrations are positively associated with body weight (20, 21). By the end of our 12-weeks study, there was no significant effect of PCSK9 overexpression on body weight in either genotype, despite continued high plasma concentrations of PCSK9 at this time point. Interestingly, in the absence of AAV-induced PCSK9 overexpression, *Dennd5b*^*−/−*^ mice on Western diet had higher plasma concentrations of PCSK9 and lower hepatic LDLR protein than wild type. While this by itself would suggest higher plasma non-HDL-C in *Dennd5b*^*−/−*^ mice, the observed effect was modest and not statistically significant. Other factors may be influencing the impact of hepatic LDLR on plasma lipid concentrations. Induction of hepatic *PCSK9* expression dramatically increased plasma PCSK9 concentrations in both genotypes, although to a lesser extent in *Dennd5b*^*−/−*^ mice and LDLR protein abundance was lowered similarly in both groups. Despite similar reductions of LDLR protein, the effects of PCSK9 on non-HDL-C concentrations were smaller in *Dennd5b*^*−/−*^ mice than wild type. The reason for reduced responsiveness to PCSK9 AAV is not clear but may be related to *Dennd5b*′s role in dietary lipid absorption or other regulatory factors that are impacting baseline PCSK9 concentrations. On the other hand, *Dennd5b*^*−/−*^ mice had higher plasma triglyceride concentrations than wild type after 12 weeks on Western diet. However, plasma triglycerides concentrations in *Dennd5b*^*−/−*^ mice were not affected by PCSK9 overexpression, whereas wild type mice experienced a 10-fold increase by week 12. These data demonstrate that *Dennd5b*^*−/−*^ mice are resistant to PCSK9-induced elevations in non-HDL-C and triglyceride. Furthermore, PCSK9 overexpression induced a drop in HDL-C concentrations in wild type mice. Although, as reported previously, *Dennd5b*^*−/−*^ mice have slightly lower HDL-C concentrations, these were not significantly affected by PCSK9 overexpression.

The effects of PCSK9 on atherosclerosis burden are consistent with the observed plasma non-HDL-C concentrations. Lesion area is strongly correlated with plasma non-HDL-C concentrations in both genotypes. This suggests that the mechanism for atheroprotection in *Dennd5b*^*−/−*^ mice is likely related to lower LDL-C elevation in response to PCSK9 AAV.

*Dennd5b*^*−/−*^ resulted in a significant effect on hepatic triglyceride and cholesterol content. However, despite the lower cholesterol content of *Dennd5b*^*−/−*^ livers, these mice had markedly increased mRNA abundance of HMG CoA reductase (*Hmgcr*), the rate limiting enzyme in cholesterol biosynthesis. This may be a compensatory mechanism in *Dennd5b*^*−/−*^ mice in response to reduced dietary cholesterol intake resulting from impaired chylomicron secretion, although reduced cholesterol absorption has not yet been demonstrated directly in these mice. Principal components analysis of global expression patterns clearly demonstrated not only that *Dennd5b* genotype impacted hepatic lipid metabolism but also that *Dennd5b*^*−/−*^ mice are resistant to the effects of PCSK9 overexpression on this set of lipid metabolism genes. Examination of individual hepatic mRNA abundances suggested reduced FA uptake and increased LD hydrolysis in *Dennd5b*^*−/−*^ mice. CD36 is a receptor which mediates uptake of long chain FAs in multiple tissues, and deletion of hepatic CD36 in mice results in protection from hepatic steatosis and inflammation (22). Lower hepatic CD36 abundance in *Dennd5b*^*−/−*^ may contribute to our observation of reduced hepatic triglyceride content. This is supported by a strong positive linear relationship between *Cd36* mRNA and hepatic triglyceride content. *Pnpla3* mRNA abundance was increased in *Dennd5b*^*−/−*^ mice relative to wild type. This is a lipid droplet-associated protein that possesses hydrolase activity toward triglycerides and retinyl esters (23). The human PNPLA3 I148M variant, which has significantly reduced triglyceride hydrolase activity, is one of the strongest genetic risk factors for hepatic steatosis (24, 25, 26). Studies in mice confirm a role for the variant in hepatic steatosis and demonstrate dynamic regulation of *Pnpla3* gene expression in response to nutrient intake and hepatic lipid content (27, 28, 29). Recent studies demonstrate that the accumulation of the I148M variant protein on hepatic lipid droplets contributes to steatosis (30). Genes involved in FA metabolism were also upregulated in *Dennd5b*^*−/−*^ mice. *Acly* catalyzes the conversion of citrate to acetyl-CoA, which can be used in de novo FA synthesis. Upregulation of this gene could be a response to increased activity of the glycolytic pathway under conditions due to the relatively low abundance of exogenous of FAs in *Dennd5b*^*−/−*^ liver. This effect could also be a response to generate precursors for cholesterol synthesis in model with low hepatic cholesterol and likely reduced exogenous cholesterol supply. Increased mRNA abundance of these genes in *Dennd5b*^*−/−*^ liver support the hypothesis that hepatic steatosis is prevented in part by altered metabolism of FAs. It is not clear what specifically is mediating these effects on hepatic lipid metabolism pathways.

mRNA abundance of several common hepatic transcriptional regulators of lipid metabolic activity were not impacted by *Dennd5b*^*−/−*^ (i.e., *Srebf1*, *Srebf2*, *Ppara*), although significantly lower mRNA abundance of *Pparg* was observed. *Pparg* is most commonly associated with regulation of lipid metabolism pathways in adipose tissue (31, 32, 33). *Pparg* is also expressed by hepatocytes and hepatic expression has been positively correlated with hepatic steatosis (34, 35). Deletion of hepatic *Pparg* can prevent hepatic steatosis, and hepatic overexpression promotes increased expression of adipogenic and FA uptake genes (34). For example, *Cd36* is a validated *Pparg* target gene in mouse adipose tissue; however, all *Pparg* target genes in different tissues have not yet been fully characterized, making it unclear if all of our observed gene effects are downstream of *Pparg* or if other regulatory elements may be involved (36). The effects on *Pparg* may also be mediated by peripheral adiposity. In mice, obesity increases hepatic *Pparg* expression and promotes FA storage (37). This may be occurring in the wild type mice in our study and to a lesser extent in *Dennd5b*^*-/-*^ mice which gain less weight. Lower hepatic expression of *Pparg* in *Dennd5b*^*−/−*^ could be a significant regulatory contributor to the lower hepatic lipid accumulation, and possible indirect modulation of this gene’s expression by *Dennd5b* could also be a component of peripheral tissue responses to *Dennd5b*^*−/−*^, particularly in adipose tissue.

A modestly elevated rate of plasma triglyceride accumulation in *Dennd5b*^*−/−*^ mice compared to wild type during the VLDL secretion assay may also contribute to lower hepatic lipid content. The lack of effect on apoB protein and possibly elevated triglyceride to apoB ratio suggests a similar number of apoB-containing particles in the plasma but with slightly higher triglyceride content per particle. This could result from secretion of VLDL particles with greater triglyceride content. Ultimately, the effect of *Dennd5b* deficiency on VLDL secretion is modest and this is likely not the mechanistic basis for lower hepatic triglyceride content in these mice. Increased VLDL-triglyceride secretion may also contribute to elevated plasma triglyceride concentrations observed in *Dennd5b*^*-/-*^ mice fed a Western diet. Although it seems likely that impaired lipid absorption due to loss of *Dennd5b* would also affect peripheral lipid uptake, the impact of *Dennd5b* on peripheral lipolysis has not been reported.

This study reveals novel insights into the impact of *Dennd5b* on hepatic and systemic lipoprotein metabolism. However, there are some limitations to the present study which need to be considered while interpreting the data. First, it is unclear if the effects of *Dennd5b*^*−/−*^ are direct on the liver or secondary to the previously reported intestinal lipid absorption phenotype. *Dennd5b* is expressed in both the liver and the intestine (as well as the brain). The mouse model utilized in these studies is a whole-body *Dennd5b*^*−/−*^. Therefore, it is difficult to distinguish direct roles for this protein in different tissues especially because of the interconnected nature of pathways mediating lipid metabolism in the intestine and liver. Future studies in cell-specific knockout mice may help to dissect the tissue-specific roles of *Dennd5b*. Another limitation is that these studies did not include comparisons to standard laboratory diet controls. While this does not diminish the magnitude of the effects observed, it does limit our ability to determine the effect size relative to “healthy” mice not consuming a Western diet. Despite these limitations, our findings support a prominent impact of *Dennd5b* expression on hepatic lipid content and plasma lipoprotein metabolism.

## Conclusion

In summary, these studies examined the impact of whole-body *Dennd5b* deficiency on PCSK9-induced effects on plasma and hepatic lipid concentrations and development of atherosclerosis. Loss of *Dennd5b* has significant effects on plasma lipoproteins in mice fed a Western diet characterized by reduced HDL-C and increased triglyceride. *Dennd5b*^*−/−*^ mice are resistant to PCSK9 effects on plasma lipoproteins and develop less atherosclerosis than wild type mice. Additionally, we demonstrated that *Dennd5b* can impact genes involved in hepatic lipid metabolism and affect hepatic lipid content suggesting a possible influence on conditions involving hepatic steatosis such as non-alcoholic fatty liver disease. It is still unclear if the protective effect of *Dennd5b* deficiency on atherosclerosis is a direct consequence of impaired absorption of dietary cholesterol or potentially a result of secondary effects of *Dennd5b* on hepatic lipid metabolism. Future studies are needed to tease out the cell-specific roles of *Dennd5b* in hepatic and intestinal lipid metabolism.

## Data Availability

The datasets generated during and/or analyzed during the current study are available from the corresponding author on reasonable request.

## Supplemental Data

This article contains supplemental data.

## Conflict of Interest

The authors have no conflicts of interest to disclose.

## Figures and Tables

**Supplemental Table S1:**